# Advancements and Perspectives in Polysaccharide-Based Nanoparticles for Theranostic Nanomedicine

**DOI:** 10.3390/ph17010036

**Published:** 2023-12-26

**Authors:** Jingdi Chen

**Affiliations:** Marine College, Shandong University, Weihai 264209, China; jdchen@sdu.edu.cn

It is with great enthusiasm that we present this Special Issue of *Pharmaceuticals*, dedicated to “Polysaccharide-Based Nanoparticles for Theranostic Nanomedicine”. This collection of nine innovative papers reflects the dynamic and rapidly evolving field of nanomedicine, specifically focusing on the use of polysaccharide-based nanoparticles.

We have seen that polysaccharides, as natural biological macromolecules with good biocompatibility, biodegradability and unique physical and chemical properties [1], not only play a therapeutic and synergistic role [2,3], but also acts as drug carriers and tools for tissue and microenvironment engineering [4,5,6]. In this Special Issue, we focus our attention on the possible mechanisms of various polysaccharides in biomodelling (contribution 3), targeted delivery (contribution 4, contribution 6, and contribution 7), and smart medical treatment (contribution 2 and contribution 8) et al. Their various applications, from synthesis and characterization to drug delivery and diagnostic imaging, will also be explored. We have witnessed the evolution of these materials from mere carriers to multifunctional systems that herald a new era in medical treatment and imaging.

In this Special Issue, the relevant mechanisms and applications of chitosan and its derivatives receive significant attention. We were delighted to see that one of the articles—Mechanism and Application of Chitosan and Its Derivatives in Promoting Permeation in Transdermal Drug Delivery Systems: A Review—was rated as a highly cited article (contribution 9). As of July/August 2023, this highly cited paper has received enough citations to place it in the top 1% of the academic field of Pharmacology and Toxicology, based on the citation threshold for publications in the field this year. This also suggests that this type of material has great potential for research and transformational applications such as wound healing (contribution 1), bone regeneration (contribution 5), transdermal drug delivery (contribution 9), and the promotion of intestinal microbiota health [7,8]. This material’s application in the field of molecular imaging should also be investigated [9,10]. We expect these articles to contribute to that process.

As we look to the future, it is clear that polysaccharide-based nanoparticles hold immense promise for advancing therapeutic and diagnostic strategies. Their adaptability and efficacy place them at the forefront of research in nanomedicine [11,12]. We are confident that this Special Issue will serve as a valuable resource for researchers and practitioners alike, and inspire further innovation in this exciting field.
